# Physical Function of RA patients Tapering Treatment—A Post Hoc Analysis of the Randomized Controlled RETRO Trial

**DOI:** 10.3390/jcm12113723

**Published:** 2023-05-28

**Authors:** Marlene Stephan, Koray Tascilar, Melek Yalcin-Mutlu, Melanie Hagen, Judith Haschka, Michaela Reiser, Fabian Hartmann, Arnd Kleyer, Axel J. Hueber, Bernhard Manger, Camille Figueiredo, Jayme Fogagnolo Cobra, Hans-Peter Tony, Stephanie Finzel, Stefan Kleinert, Jörg Wendler, Florian Schuch, Monika Ronneberger, Martin Feuchtenberger, Martin Fleck, Karin Manger, Wolfgang Ochs, Matthias Schmitt-Haendle, Hannes Martin Lorenz, Hubert Nüsslein, Rieke Alten, Joerg Henes, Klaus Krüger, Georg Schett, Jürgen Rech

**Affiliations:** 1 Department of Internal Medicine 3, FAU Erlangen-Nuremberg and Universitätsklinikum Erlangen, 91054 Erlangen, Germany; marlenestephan@gmx.de (M.S.); melek.yumermutlu@uk-erlangen.de (M.Y.-M.); fabian.hartmann@uk-erlangen.de (F.H.); arnd.kleyer@uk-erlangen.de (A.K.); axel.hueber@klinikum-nuernberg.de (A.J.H.); bernhard.manger@uk-erlangen.de (B.M.); georg.schett@uk-erlangen.de (G.S.); 2 Deutsches Zentrum fuer Immuntherapie (DZI), FAU Erlangen-Nuremberg and Universitätsklinikum Erlangen, 91054 Erlangen, Germany; 3 Karl Landsteiner Institute for Gastroenterology and Rheumatology, 1100 Vienna, Austria; judith.haschka@bhs.at; 4 Ludwig Boltzmann Institute of Osteology, I Medical Department, Hanusch Hospital Vienna, 1140 Vienna, Austria; 5 Institutio de Rheumatologia, Sao Paolo 01317-001, Brazil; figueiredocamille@gmail.com (C.F.); jayme.cobra@gmail.com (J.F.C.); 6 Rheumatology/Clinical Immunology, Department of Internal Medicine II, University of Würzburg, 97080 Würzburg, Germany; tony_h@ukw.de; 7 Department of Rheumatology and Clinical Immunology, Medical Center-University of Freiburg, Faculty of Medicine, University of Freiburg, 79106 Freiburg, Germany; stephanie.finzel@uniklinik-freiburg.de; 8 Rheumatology Clinical Practice Erlangen, 91054 Erlangen, Germanyjoerg.wendler-erlangen@t-online.de (J.W.); florian.schuch@pgrn.de (F.S.); monika.ronneberger@pgrn.de (M.R.); 9 Rheumatology Practice and Department of Internal Medicine 2, Clinic Burghausen, 84489 Burghausen, Germany; rheumatologie@med-bayern-ost.de; 10 Asklepios Medical Center, Department of Rheumatology and Clinical Immunology, 93077 Bad Abbach, Germany; 11 Rheumatology Practice Bamberg, 96047 Bamberg, Germany; 12 Rheumatology Practice Bayreuth, 95444 Bayreuth, Germanyschmitms@me.com (M.S.-H.); 13 Department of Medicine V, Center for Rheumatic Diseases Baden-Baden, University Hospital Heidelberg, 69120 Heidelberg, Germany; hannes.lorenz@med.uni-heidelberg.de; 14 Rheumatology Practice Nürnberg, 90429 Nürnberg, Germany; 15 Schlosspark Klinik, Internal Medicine/Rheumatology, 14059 Berlin, Germany; 16 Centre for Interdisciplinary Clinical Immunology, University of Tübingen, 72076 Tübingen, Germany; 17 Praxiszentrum St. Bonifatius, 81541 Munich, Germany; klaus.krueger@med.uni-muenchen.de

**Keywords:** HAQ, Rheumatoid Arthritis, PROM’s, DMARD, DAS28

## Abstract

Several studies have shown that tapering or stopping disease-modifying anti-rheumatic drugs (DMARDs) in rheumatoid arthritis (RA) patients in sustained remission is feasible. However, tapering/stopping bears the risk of decline in physical function as some patients may relapse and face increased disease activity. Here, we analyzed the impact of tapering or stopping DMARD treatment on the physical function of RA patients. The study was a post hoc analysis of physical functional worsening for 282 patients with RA in sustained remission tapering and stopping DMARD treatment in the prospective randomized RETRO study. HAQ and DAS-28 scores were determined in baseline samples of patients continuing DMARD (arm 1), tapering their dose by 50% (arm 2), or stopping after tapering (arm 3). Patients were followed over 1 year, and HAQ and DAS-28 scores were evaluated every 3 months. The effect of treatment reduction strategy on functional worsening was assessed in a recurrent-event Cox regression model with a study-group (control, taper, and taper/stop) as the predictor. Two-hundred and eighty-two patients were analyzed. In 58 patients, functional worsening was observed. The incidences suggest a higher probability of functional worsening in patients tapering and/or stopping DMARDs, which is likely due to higher relapse rates in these individuals. At the end of the study, however, functional worsening was similar among the groups. Point estimates and survival curves show that the decline in functionality according to HAQ after tapering or discontinuation of DMARDs in RA patients with stable remission is associated with recurrence, but not with an overall functional decline.

## 1. Introduction

Rheumatoid arthritis (RA) is associated with progressive disability and early death and requires lifelong therapy with disease-modifying drugs (DMARDs), ultimately inflicting a high socioeconomic burden [1]. Over the years, treatment of RA has substantially evolved, and many RA patients experience remission of disease due to earlier intervention, frequent monitoring, and treatment-adjustment guided by stringent disease activity targets and the development of highly effective targeted treatments. These achievements, along with the changing disease definition [2], led to a considerable RA patient population which does not show symptoms of disease but is taking continuous DMARD treatment [2]. This growing population of RA patients under remission poses an important challenge since the need to broaden the evidence for the management of remission in RA becomes more apparent.

We have previously reported analyses from the RETRO study showing that tapering and stopping DMARDs in RA patients in sustained remission is feasible [3] and lowers costs [4]. However, flares of RA are more frequent when tapering and stopping DMARDs [3]. While remission or low disease activity can still be restored after re-starting the previous DMARD treatment [5], flares are hard to predict and may lead to joint damage and worsening functional status over time [6,7]. Therefore, it is necessary to understand the impact of treatment reduction or cessation not only on disease activity but also on functional status in order to better inform the patient and to develop better strategies for remission management. To this end, we performed a post hoc analysis of data from the RETRO trial to characterize the change in functional status during RETRO trial interventions.

## 2. Methods

### 2.1. Study Design and Patients

RETRO was a phase 3, open-label, parallel-group, multicenter, randomized-controlled study (EudraCT number: 2009-015740-42). Patients had to be in sustained Disease Activity Score using 28 joints (DAS28) with erythrocyte sedimentation rate (ESR) remission (score < 2·6 units) at randomization, documented in at least three consecutive visits during the previous 6 months [3]. Reduction was performed as follows: Conventional DMARDs, corticosteroids, and intravenous tocilizumab were achieved by reducing an individual patient’s dose by 50% without changing the dosing intervals. Reduction of tumor necrosis factor (TNF) inhibitors and abatacept was done by doubling the dosing intervals. Non-steroidal anti-inflammatory drugs could be taken on demand before and after inclusion in the study [3]. Further details are described elsewhere [3,8]. The primary objective of the study was to assess the possibility of treatment reduction and discontinuation in RA patients under sustained remission. The study was approved by the local ethics committee and conducted according to the guidelines of the Declaration of Helsinki. Written informed consent was obtained from each participant before screening.

### 2.2. Outcome

Health Assessment Questionnaire (HAQ) data were collected at baseline and 3, 6, 9, and 12 months. The outcomes for this analysis were HAQ at study visits, HAQ change from baseline, and functional worsening defined as an HAQ increase of ≥0.25 corresponding to a commonly used minimal clinically important change threshold [9].

### 2.3. Statistical Analysis

We tabulated baseline patient characteristics using appropriate summary statistics for continuous and categorical data. The RETRO trial was powered based on the expected flare rates in study groups, and a separate power analysis was not conducted for HAQ worsening, since this is a post hoc analysis [10]. We used the Kaplan–Meier method to summarize the risk of HAQ worsening over time in study groups and used the log-rank test of trend for an overall comparison of HAQ-worsening-free survival. Cox regression was used to estimate the hazard ratio (HR) of HAQ worsening over time. A linear mixed effects regression was fit to analyze the mean HAQ across study groups. This analysis used HAQ measurements obtained across all visits as the dependent variable, a categorical variable indicating the visit and study group and an interaction term for visits and study groups. A categorical participant identifier variable was used as the cluster identifier for random intercepts. A 95% confidence interval for the visit-study group interaction term excluding zero was considered to indicate a significant difference of mean HAQ between groups for a given visit. Otherwise, two-tailed P values less than 0.05 were considered significant. Analyses were conducted using the open-source R software v. 4.0.1 (R Foundation for Statistical Computing, Vienna, Austria) working under the GUI RStudio v. 1.2.1 (Rstudio, Boston, MA, USA). No specific procedure was utilized for handling missing HAQ data.

## 3. Results

### 3.1. Patients

One-year follow-up data of 282 patients enrolled in the RETRO study were available. The patients were randomized 1:1:1 in the three different treatment arms: arm 1 (control, n = 93), arm 2 (tapering, n = 93), and arm 3 (tapering and stopping, n = 96). Table 1 shows the baseline characteristics of the patients. Mean age was 56.5 (±13.0) years, mean disease duration was 7.4 (±7.3) years, and 59.4% (n = 167) were female. All patients were in sustained clinical remission with a mean DAS28-ESR score of 1.7 (±0.6) at baseline. The mean HAQ at baseline over all three groups was 0.2 (±0,4) and identical among the three groups. The low HAQ score at baseline indicated good function of RA patients in sustained remission. There were 75.9% of patients taking csDMARD treatment with methotrexate (n = 214), while 43.6% (n = 123) of patients were taking bDMARD, most of them TNF inhibitors.

### 3.2. Physical Function over Time

When analyzing mean HAQ values at baseline and 3, 6, 9, and 12 months after the intervention, we found that physical function was equally good throughout the observation time and virtually identical among the three study arms. Thus, mean HAQ at study visits ranged between 0.14 (0.30) and 0.21 (0.38) units among the three study groups across all visits. Notably, values were well below HAQ values of 0.5, which means that there is no relevant disability.

**Figure 1:**
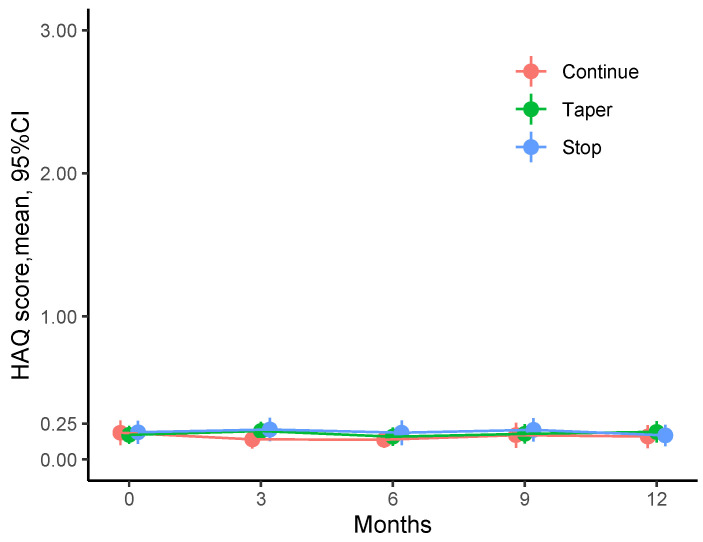
Physical function measured with the HAQ (Health assessment questionnaire) during the tapering of DMARDs (disease-modifying antirheumatic drug ) in RA (Rheumatoid Arthritis)patients in stable re-mission.

The change from baseline HAQ at study visits ranged between −0.052 (0.292) and 0.032 (0.201), and all mean change values were below the minimal clinically important change threshold of 0.25. Figure 1 summarizes the mean HAQ values and changes from baseline for each study group and visit. As described by Tascilar et al., 173 (61%) of 282 patients remained in DAS28 remission without any relapse during the study period. One-hundred and nine (39%) had relapses. Crude proportions of relapses were 16 (17%) of 93 patients for continue, 40 (43%) of 93 for taper, and 53 (55%) of 96 for stop [3].

Mean HAQ (Health assessment questionnaire) values were measured for each treatment arm (continuation, tapering and discontinuation of medication) and each visit (every 3 months). Average HAQ values were less than 0.25 units throughout the study.

### 3.3. Incident Decline in Physical Function

**Figure 2:**
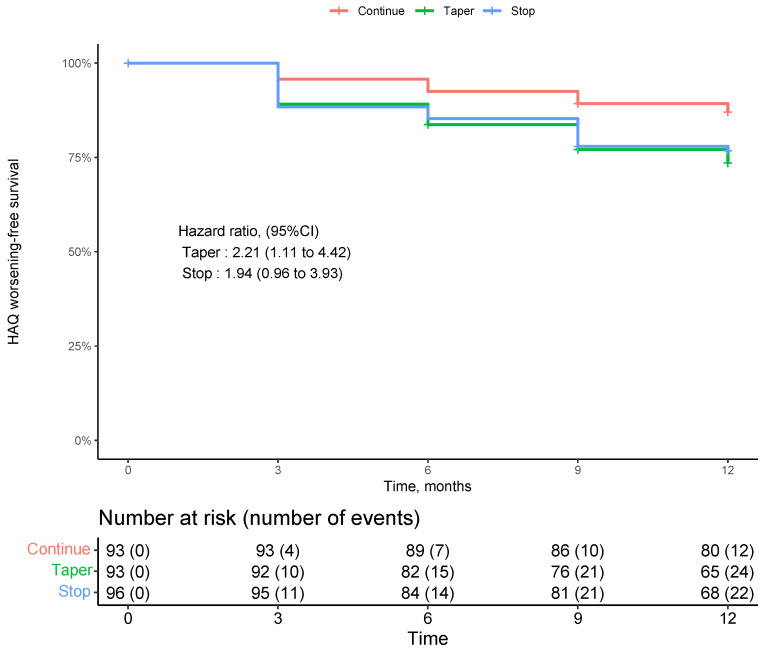
Incident functional worsening during tapering of DMARDs (disease-modifying antirheumatic drug’s in RA (Rheumatoid Arthritis) patients in stable remission.

Although the mean HAQ values and change values were low, we performed a survival analysis for the risk of functional decline by study intervention. Two-hundred and eighty-two patients with at least one post-baseline follow-up were included in this analysis. Overall, incident functional impairment was rare as shown in Figure 2. Survival curves showed a numerically higher incident decline in physical function in the tapering and stopping groups. However, this difference was not across study groups (*p* = 0.87 by log rank test of trend).

The survival analysis was performed for the risk of functional decline for each treatment arm (continuation, tapering and discontinuation of therapy). Two-hundred and eighty-two patients with at least one post-baseline follow-up were included in this analysis. We did not find a significant difference of functional decline over time across study groups, while there was a trend to higher incident functional worsening in those patients tapering DMARDs.

## 4. Discussion

Reducing DMARD therapy becomes an option for RA patients in sustained remission. The possibility for reducing treatment in stable remission has been included into the ACR and EULAR criteria of management of RA [2,11]. Still, the question is whether and under which conditions a patient with RA in stable remission could taper or stop DMARD therapy or whether treatment should be continued. Stopping DMARD therapy, of course, has the advantage of reducing adverse events and costs [4,12]. While relapse rates were higher in patients tapering or stopping DMARDs, RETRO showed that more than half of them stayed in remission despite DMARD tapering [3,8]. This suggested defining the accurate patient profile for tapering or even stopping DMARDs. Firstly, tapering or stopping therapy should only be considered in patients with very low or complete absence of disease symptoms [13]. Additionally, our previous data revealed that serological examination can help in predicting the relapse risk in patients tapering DMARD [14]. J. Symptom duration at presentation and the absence of RA-specific autoantibodies are associated with sustained DMARD-free remission [15].

Keeping good physical function is key to RA treatment and of interest for the patient. Hence, tapering or even stopping DMARD treatment should not put the patients at risk for deteriorating physical function. If tapering or stopping DMARD therapy does not worsen the physical function of the patient, it might help to advocate the reduction of DMARDs in stable remission and in consequence reduce potential overtreatment and adverse events. Herein, we show that physical function does not deteriorate with the tapering of DMARDs. This observation can be explained by the fact that in case of flare of RA during DMARD tapering, effective DMARD therapy was re-installed and the patients responded well to this treatment and thereby improved in function. Hence, overall physical function remained good during the follow-ups. Flares may indeed have been the reason for spurious impairment of function during the trial, which was evident from the survival curves, showing that the tapering arms, which are characterized by higher flare rates, were more prone to lose, though spuriously, their functional remission status.

The limitations of the study are due to the fact that the data on function are limited to one year. Hence, it cannot be completely ruled out that after one year, there might be still a loss in physical function. In addition, physical function in the RETRO study was patient-reported and did not include any objective measurements such as grip strength or hand function. However, we think this is unlikely as patients who had flared were exposed to effective treatment and regained functional remission. Another limitation is that we used stable DAS28 of less than 2.6 units as an inclusion criterion allowing tapering or stopping DMARDs. ACR guidelines state that patients‘ medication should not be tapered unless the patient is in ACR/EULAR remission [11]. However, most of these patients also fulfilled ACR/EULAR remission and were in deep remission for over a year upon inclusion [3]. Hence, we think that this population is a representative RA population, in which treatment DMARD tapering is considered. Finally, data for structural damage progression, i.e., radiographic scores, were not collected, although patients in remission with good physical function might show dysfunctional joints or increased joint damage [16,17,18].

## 5. Conclusions

Point estimates and survival curves show no decline in physical function after tapering or stopping DMARDs in RA patients with stable remission. While there is a tendency to a higher incidence of impaired physical function due to flares in the tapering groups, no overall decline in physical function was observed as RA patients experience flares returned to their activate drug treatment and regained functional remission.

## Figures and Tables

**Table 1:** Baseline-Characteristics.

	Group			
	Continue	Taper	Stop	Overall
N	93	93	96	282
Age, mean Standard Deviation (SD)	55.9 (12.7)	56.9 (13.0)	56.5 (13.3)	56.5 (13.0)
Female, n (%)	53 (57.0)	57 (62.0)	57 (59.4)	167 (59.4)
Rheumatoid Factor (RF), n (%)	52 (55.9)	58 (62.4)	52 (54.2)	162 (57.4)
Anti-citrullinated protein antibodies (ACPA`s), n (%)	53 (57.0)	50 (54.9)	55 (57.3)	158 (56.4)
Disease duration, years, mean (SD)	7.6 (6.9)	7.8 (6.9)	6.8 (8.1)	7.4 (7.3)
Remission duration, months, mean (SD)	20.6 (18.0)	16.5 (15.9)	22.7 (30.4)	20.0 (22.6)
Health Assessment Questionnaire (HAQ), standard, mean (SD)	0.2 (0.4)	0.2 (0.3)	0.2 (0.4)	0.2 (0.4)
DAS28, mean (SD)	1.7 (0.7)	1.7 (0.6)	1.7 (0.6)	1.7 (0.6)
Glucocorticoids, n (%)	27 (29.0)	23 (24.7)	17 (17.7)	67 (23.8)
csDMARD (conventional synthetic disease - modifying antirheumatic drug —total, n (%)	85 (91.4)	81 (87.1)	84 (87.5)	250 (88.7)
csDMARD—solo, n (%)	47 (50.5)	46 (49.5)	52 (54.2)	145 (51.4)
csDMARD—combination, n (%)	6 (6.5)	3 (3.2)	5 (5.2)	14 (5.0)
Methotrexate, n (%)	72 (77.4)	67 (72.0)	75 (78.1)	214 (75.9)
Antimalarials	14 (15.1)	10 (10.8)	6 (6.2)	30 (10.6)
Leflunomide	5 (5.4)	5 (5.4)	8 (8.3)	18 (6.4)
Sulfasalazine	4 (4.3)	4 (4.3)	2 (2.1)	10 (3.5)
Azathioprine	1 (1.1)	1 (1.1)	0 (0.0)	2 (0.7)
Biologics—total, n (%)	40 (43.0)	44 (47.3)	39 (40.6)	123 (43.6)
Biologics—solo, n (%)	8 (8.6)	12 (12.9)	12 (12.5)	32 (11.3)
Biologics—combination, n (%)	32 (34.4)	32 (34.4)	27 (28.1)	91 (32.3)
anti-TNF, n (%)	28 (30.1)	33 (35.5)	29 (30.2)	90 (31.9)
Adalimumab	8 (8.6)	13 (14.0)	11 (11.5)	32 (11.3)
Certolizumab	7 (7.5)	10 (10.8)	8 (8.3)	25 (8.9)
Etanercept	7 (7.5)	4 (4.3)	7 (7.3)	18 (6.4)
Infliximab	6 (6.5)	4 (4.3)	3 (3.1)	13 (4.6)
Golimumab	1 (1.1)	2 (2.2)	0 (0.0)	3 (1.1)
Other biologics, n (%)	11 (11.8)	11 (11.8)	10 (10.4)	32 (11.3)
Tocilizumab	10 (10.8)	9 (9.7)	8 (8.3)	27 (9.6)
Abatacept	1 (1.1)	2 (2.2)	2 (2.1)	5 (1.8)

## Data Availability

Deidentified participant data collected for the study, including individual participant data and a data dictionary defining each field in the set, will be made available to others upon request (email koray.tacilar@ukerlangen.de). The study protocol, statistical analysis plan, and informed consent form are also available from koray.tacilar@uk-erlangen.de.
